# Reliability and screening ability of the StarT Back screening tool in patients with low back pain in physiotherapy practice, a cohort study

**DOI:** 10.1186/s12891-017-1553-x

**Published:** 2017-05-31

**Authors:** Hilde Stendal Robinson, Hanne Dagfinrud

**Affiliations:** Department of Health Sciences, Institute of Health and Society, University of Oslo, P.O. Box 1089, Blindern 0317 Oslo, Norway

**Keywords:** Low back pain, Physical function, Reliability, Validity, Test-retest, Physiotherapy, Primary health care, Prognostic indicators

## Abstract

**Background:**

Low back pain (LBP) is the most common reported musculoskeletal disorder, with large prevalence numbers and high costs. Focus on early identification of patients at risk of developing chronic LBP has increased. The Keele Start Back Tool (SBT) is a questionnaire aiming at screening prognostic indicators in LBP patients, categorizing patients into risk-groups and guide treatment. The aim of this study was to explore the Norwegian version of the SBT with regard to reliability of the SBT-scoring and the screening ability in LBP patients in primary care physiotherapy.

**Methods:**

LBP patients answered a package of questionnaires twice, with 1-3 days in between, containing SBT, Hannover functional ability questionnaire, pain intensity questions and demographics. The relative and absolute reliability of SBT was calculated using intraclass correlation coefficient (ICC) and the smallest detectable change respectively. Independent sample t-tests were used for group comparisons.

**Results:**

Fifty-two patients with LBP. Mean age (SD) was 45 (12) years and 62% were female. The ICC (95% CI) for SBT total score and psychosocial subscore was 0.89 (0.82, 0.94) and 0.82 (0.70, 0.90) respectively. None of the participants were allocated to the high risk group. The medium risk group reported significantly more pain last week and more activity limitations than the low risk group at both test and retest (0.001 ≤ p ≤ 0.003), whereas no significant difference between the groups was found on pain now (0.05 ≤ p ≤ 0.16).

**Conclusions:**

The Norwegian version of the SBT was reliable and the screening ability was good as the subgrouping of patients into risk-groups reflected the severity of their back problems. The SBT may be an applicable and useful tool in physiotherapy practice.

## Background

Low back pain (LBP) is reported to be the most common musculoskeletal disorder, with large costs for patients and society [1]. Life-time prevalence for LBP has been reported to be between 60 and 85% [2–5] and 34% of the participants in a large population study in Norway reported to have had LBP last week [6]. Diagnosing LBP is challenging and the cause is often unclear. Due to the lack of diagnostic tests that can identify objective signs of the condition, most of the patients are characterized as having “non-specific LBP” [7, 8]. Most patients with acute LBP are reported to recover within 6 weeks, but symptoms remain in about 5 to10%, and this proportion is at risk for developing chronic LBP [1].

Several factors are shown to be associated with the risk of non-recovery from LBP, such as personal factors (age, gender, general health), work-related factors, radiating or widespread pain and psychosocial factors [5, 9–15]. Psychosocial factors are shown to be important indicators for chronicity and development of disability due to musculoskeletal pain [16]. In the last decade, there has been an increased focus on early identification of patients at risk for developing persistent LBP. Different screening tools have been launched to facilitate identification of prognostic psychosocial factors, so-called “yellow flags”. An example is the Keele Start Back Tool (SBT) which is a brief questionnaire for screening prognostic indicators (both physical and psychosocial risk factors) for persistent, disabling back pain. Based on the SBT-scores, patients can be categorized into three subgroups: patients with low, medium or high risk for developing persistent LBP and activity limitations [17–19]. According to current treatment recommendations, the low risk group should receive minor attention from health professionals, and self-management strategies are recommended for these patients. The medium risk group should be offered physiotherapy, and for the high risk group more psychologically informed interventions are recommended [17, 18, 20].

The SBT subgroups may provide useful information for optimal allocation of patients to appropriate treatment. To be useful as a screening tool in physiotherapy practice, it is important that the SBT-scoring is reliable and that the allocation to risk groups reflects the severity of the patients’ back problems. We hypothesized that patients categorized as low risk should have less pain and less perceived activity limitations than patients categorized in the moderate or high risk groups. Further, we expected that few of the patients seeing physiotherapists in primary health care would be categorized into the high risk group. Thus, the aim of this study was to explore the Norwegian version of the SBT with regard to reliability and screening ability in LBP patients in primary care physiotherapy.

## Methods

This paper is based on data from a larger study also examining clinical tests for LBP patients [21]. Patients with LBP, treated by physiotherapists (PT) in Oslo (capital) and Hedmark (county), Norway, were invited to participate in the study by their PT. The only inclusion criterion was, besides seeking physiotherapy for LBP, age between 18 and 70 years old. Two PTs were responsible for the administration of the questionnaires and the clinical examinations, and 52 patients were consecutively recruited during 1 year (2014).

### Procedure

Each participant filled in the package of questionnaires and was examined clinically twice with 1 – 3 days in between. The present study is based only on data from the questionnaires. The questionnaires included demographic variables (gender, age, height, weight, work situation, daily activity level), pain intensity, now and last week (VAS, 0–10, where 10 is worst pain), as well as the SBT and the Hannover functional ability questionnaire (Hannover), which captures the patients’ perceived capability to perform daily activities [20]. At retest, the patients were also asked if their back pain had changed since the last examination (answer alternatives: much better, better, the same, worse, much worse).

### Measurements

The SBT consists of 9 items; referred leg pain, comorbid pain, disability (2 items), bothersomeness, catastrophizing, fear, anxiety, and depression. All questions are answered by agree (1) or disagree (0), except the question regarding bothersomeness “Overall, how bothersome has your back been the last two weeks?” which is answered on a 5 point Likert scale with response alternatives: not at all (0), slightly (0), moderately (0), very much (1) and extremely (1) (bothersome). Hence, the total score range from 0 to 9, with 9 indicating worst prognosis. The last 5 items are summarized into a psychosocial subscale with 5 as the maximal score, indicating high risk for development of chronic LBP [17]. Patients with total score between 0–3 are classified as low risk (minimal treatment, e.g. self-management strategies), those scoring a minimum of 4 points on total score of which a maximum of 3 items from the psychosocial risk factors are classified as medium risk (appropriate for physiotherapy management) and those scoring 4 or 5 on the psychosocial subscale are classified as high risk of poor prognosis regarding persistent disability (suitable for management with psychologically informed interventions) [22].

The Hannover questionnaire contains 12 questions about the ability to perform daily activities such as washing and drying one self, putting on socks, lifting objects, running, long time standing and longtime sitting [20]. Each item is scored on a 3-point ordinal scale (0 = yes, can perform the task without difficulties, 1 = yes, can perform task with difficulties, 2 = no, can perform task only with help from others). A sum score of all items is made, reaching from 0 to 24, with 0 representing the ability to perform the tasks without difficulties and 24 representing not having the ability to perform the tasks without getting help. Magnussen and co-workers (2010) reported satisfactory psychometric properties of the Norwegian version of the Hannover questionnaire [20]

The study procedures were carried out according to the Helsinki Declaration and were approved by the Regional Committee for Medical Research Ethics, Norway (2013/2030). All participants gave written informed consents.

### Statistical analysis

Descriptive data were used to characterize the study sample and presented as frequencies, percentages, or means with standard deviations (SD). The distribution of pain and activity limitations was presented for each SBT subgroup. Independent sample t-tests were used for group comparisons, and the results were presented with mean difference and 95% confidence interval (CI).

The relative reliability between the SBT scores at test and retest was calculated using intraclass correlation coefficient (ICC_3,1_) with 95% CI. We categorized the results as follows: ICC < 0.40 as poor reliability, 0.40 ≤ ICC < 0.75 as good reliability and ICC ≥ 0.75 as excellent reliability [23]. Furthermore, Bland and Altman plots were used to evaluate limits of agreement between test and retest [24]. The absolute reliability [25] was presented as the smallest detectable change (SDC) using the standard error of measurement SEM = SD_diff_/√2 and the SDC = 1,96*√2 *SEM. SDC reflects the smallest change in score that can be interpreted as change above measurement error in an individual.

Patients were excluded from the test-retest analyses if they reported their back pain to be much better or much worse at retest.

Floor and ceiling effects for the SBT total score were measured by calculating the proportion of participants with scores within the measurement error from worst score (worst score - SDC) and best score (best score + SDC). A fraction of 15% or more was considered as floor or ceiling effect [26].

Internal consistency was measured by Cronbach’s alpha (α) for the total score and the psychosocial subscore. Poor internal consistency was defined as α < 0.7, item redundancy was defined as α < 0.9.

Data analysis was performed using SPSS version 22.0 (IBM Corp., New York, NY) and a 5% level of significance was used.

## Results

A total of 52 participants were included, 45 (SD 12) years old and 32 (62%) were women. Eleven percent with LBP in 12 weeks or less and 64% with LBP in more than 12 months, 48% worked full time (Table 1). Mean (SD) total score on the SBT was 2.9 (1.7) and 2.9 (1.8) on test and retest respectively, and similarly 0.9 (1.1) and 0.8 (1.1) for the psychosocial subscore. The patients were allocated into risk-groups based on the SBT-scores at the first test as follows: 31 (60%) low risk, 21 (40%) medium risk and none (0%) high risk.

**Table 1:** Description of participants

*N* = 52		Mean (SD)	Frequency (%)
Age		45 (12)	
Gender, female			32 (62)
Duration LBP	0–12 weeks		6 (11)
	13–26 weeks		5 (11)
	27–52 weeks		7 (14)
	>52 weeks		33 (64)
Working situation	Full time work		25 (48)
	100% sick leave		3 (6)
	100% disability benefit		9 (17)
	Graded sick leave		8 (15)
	Other		6 (11)
Pain intensity	Now	3.4 (2.0)	
	Last week	5.6 (2.2)	

*SD* standard deviation, *LBP* low back pain

### Allocation into risk groups

The medium risk group reported significantly more *pain last week* and more *activity limitations* than the patients in the low risk group at both test and retest (0.001 ≤ p ≤ 0.003), whereas no significant difference between the groups was found on *pain now* at both test and retest (0.05 ≤ p ≤ 0.16) (Table 2). Since none of the participants were allocated to the high risk group, no comparison with this group could be made. The mean differences (95% CI) between the medium and low risk group for *pain now*, *pain last week* and *function* (Hannover) at the first test was 1.2 (0.0, 2.3), 1.9 (0.7, 3.1) and 4.8 (2.5, 7.0) respectively (Table 2).

**Table 2:** Comparison of pain intensity and functional ability (Hannover) in the Keele Start Back Tool (SBT) low and medium risk groups

N = 47	SBT classification		
	Low risk (*n* = 28)	Medium risk (*n* = 19)	*p*-value*
Test			
Pain now, mean (SD)	3.0 (1.8)	4.2 (2.1)	0.05
Pain last week, mean (SD)	4.6 (1.8)	6.6 (2.2)	0.002
Functional ability			
(Hannover), mean (SD)	6.0 (3.8)	11.0 (3.6)	<0.001
Retest			
Pain now, mean (SD)	3.4 (1.6)	4.2 (2.1)	0.16
Pain last week, mean (SD)	4.6 (1.9)	6.6 (2.5)	0.003
Functional ability			
(Hannover), mean (SD)	5,9 (4.3)	10.0 (4.0)	0.001

*Hannover*, Hannover functional ability questionnaire, *SBT* Start Back Tool, *SD* standard deviation*: independent *t*-test

### Reliability

Five participants (9.6%) reported their back pain to be *much better* or *much worse* on retest and were excluded from the reliability analyses.

**Fig. 1:**
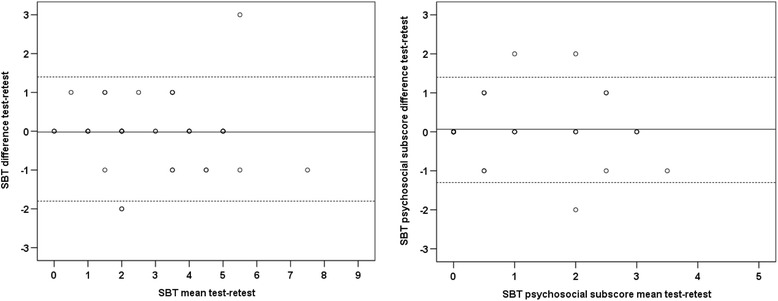
Bland Altman plot representation of the test-retest reliability, the difference against the mean of the Keele Start Back Tool (SBT) total score (left) and psychosocial subscores (right). Mean of the difference ± standard deviation was -0.2 ± 0.8 and 0.07 ± 0.7 for total score and psychosocial subscore respectively. The solid line represents mean difference. Dotted line represents limits of agreement (LoA) = mean difference ± 1.96 x SD_diff_ (-0.2 ± 1.96x0.8 = -1.8, 1.4 and 0.07 ± 1.96x0.7 = -1.3, 1.4 for the total score and the psychosocial subscore respectively)

The ICC_3,1_ (95% CI) for the SBT total score and psychosocial subscore was 0.89 (0.82, 0.94) and 0.82 (0.70, 0.90) respectively, showing that the relative test-retest reliability was excellent. Absolute reliability measured by SDC was 1.60 and 1.35 for the total score and the psychosocial subscore respectively. The Bland Altman plots revealed no systematic bias (Fig. 1).

**Fig. 2:**
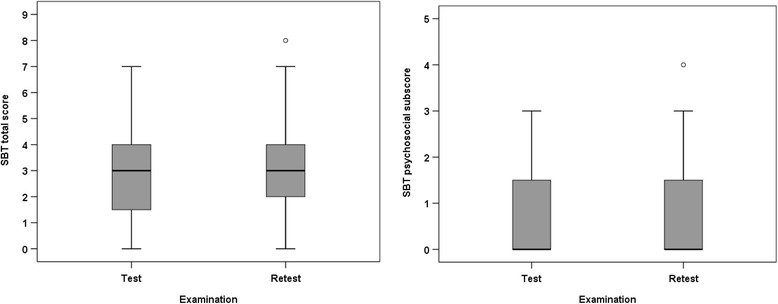
Box plots showing the Keele Start Back Tool (SBT) total score at test and retest (left) and psychosocial subscore at test and retest (right). Median, quartiles and range are shown

Nineteen percent of the participants scored below 1.60 (0 + SDC) on total score, hence the measurement of their improvement could be hampered [26]. None scored above 7.4 (9-SDC) on total score (i.e scoring of worsening could be hampered [26]). For the psychosocial subscore no floor or ceiling effects were found. Three (6.4%) and four (8.5%) participants scored 0 (lowest) on the SBT total score at test and retest respectively and none scored highest (9) (Fig. 2).

Chronbach’s alpha, (95% CI) for the total score and the psychosocial subscore was 0.51 (0.28 – 0.69) and 0.58 (0.37 – 0.74) respectively, hence the internal consistency was poor. We also performed if-item-deleted analyses, and found that for total score the removal of question 2 increased alpha to 0.61. No other question influenced the alpha. Furthermore, for the psychosocial subscore removal of question 5 increased alpha to 0.61. However, the corrected-item-total was low for both questions, -0.14 and 0.13 respectively.

## Discussion

In this study of patients with LBP in primary care physiotherapy practice, patients categorized as having medium risk for developing persistent LBP reported significantly more pain and activity limitations than patients categorized as having low risk. None of the participants were categorized as having high risk for developing persistent LBP and activity limitations. Further, the Norwegian version of the SBT was reliable.

The aims of treatment of LBP patients often include actions to reduce pain and to improve activities and participation [27]. The difference in pain and activity limitations between the low and the medium risk groups supports the suggestions that the groups should be offered different treatment. No participants were allocated into the high risk group in this study, in contrast to what has been reported previously [19, 28, 29]. The reason for this may be that our participants were recruited from patients treated by physiotherapists (PT) in primary health care, while others have recruited patients from general practices (GP) [29] and from both GP and PT [30]. According to Hill and Co-workers (2008), the high-risk group should be offered more psychologically informed interventions [17]. Thus, a reason for the few high-risk patients included in this study can be that they have been referred to other health professionals or treatment alternatives than physiotherapy. It could also be that the physiotherapists failed or avoided to recruit the high risk patients. Unfortunately, we have no information to support this and we have no information regarding patients invited and declining to participate in the present study. Thus, the selection of participants may have been biased of reasons unknown to the researchers.

Previous studies have compared the SBT with the results from the original study [29] and the frequently used Roland Morris disability questionnaire [28], a validated instrument measuring performance of activities. It has been discussed whether it is most important to reveal the patients’ performance of a task, or his *capability* of performing the task or activity [31]. The capability is considered to be influenced by the patients’ perceived self-efficacy or the belief that he really can perform the task. The Hannover questionnaire asks for the patients’ perceived ability to perform daily activities, which is useful information to physiotherapists for the screening of risk factors for persistent problems.

In contrast to previous studies [17, 28, 29], we found that in 19% of the participants in our study, the measurement of their improvement on the SBT total score could be hampered, since they already scored so close to the best score. However, our criterion on floor and ceiling effects was stricter than the criteria used in the mentioned studies. When using the same criteria (i.e. that 15% scoring the actual worst or best value) [32] we found that only 6.4% scored best (0) and none scored worst (9) on the SBT total score on test, indicating no floor or ceiling effects according to this criteria. Consequently, the SBT may have potential to measure both improvement and deterioration in this population. Cronbach’s alpha indicated poor internal consistency, and was lower than reported in the original SBT study [17]. It is, however, not unusual that measurement tools perform better in the developmental study than in the following studies [33] and our results are in accordance with results from other studies [29, 34]. The if-item-deleted analysis showed only small effects on alpha, we found an increase from 0.51 (total score) and 0.58 (psychological subscore) to 0.61 on both scores. It has previously been suggested that due to low internal consistency, the psychosocial subscore could better be seen as an index of different psychosocial constructs than as one overall distress factor [29]. However, this suggestion was not based on results from if-item-deleted analyses. Our findings appear to support this suggestion.

The total group of patients with LBP is heterogeneous and classification into subgroups is complicated. In a recent study exploring trajectories in LBP, the authors underline that LBP typically is characterized by episodic course, and reporting the duration of symptoms may therefore be difficult [35]. In the present study, most of the participants (29, 62%) have had LBP for more than 1 year, and only 5 (11%) for less than 3 months. However, all participants were included when visiting physiotherapists for a new episode of back pain, and screening for yellow flags may be relevant also when patients have experienced episodes of LBP previously. Even though our sample included more patients with long-term LBP than previous studies of the SBT [17, 29, 30] the patients in our study are representative of the LBP patients treated by physiotherapists in primary care in Norway [36, 37]. This suggests that the SBT can be used on LBP patients in different phases of the condition.

Our study is hampered with some limitations. The number of participants can be seen as relatively low, and the statistical power is therefore limited. By including only the stable participants in the reliability analyses, the number was just below the recommendations of 50 participants for this type of methodological studies [38]. However, previous studies on the SBT have larger numbers included in their study sample, but yet lower numbers in the test-retest analyses [29, 34]. Furthermore, the recruitment of patients from different geographical outpatient clinics as well as the wide inclusion criteria contributes to increase the external validity of the study. The heterogeneity among the patients concerning pain intensity, pain duration, pain distribution and sick leave makes them representative for LBP patients treated by physiotherapists in primary health care.

The short interval between test and retest increases the possibility of recall bias in the present study. However, as the study protocol included a diversity of clinical tests and questionnaires, the risk for recall bias have probably been moderate. On the other side, the short interval increased the chance that the patients’ condition was stable, which is crucial for the assessment of the reliability of an instrument. Furthermore, it was important to minimize the delay of the treatment startup for the participants.

## Conclusions

The findings in this study indicate that the SBT is reliable and the screening ability was good as the subgrouping of patients into the different risk-groups reflected the severity of their back problems. Hence, the SBT may be an applicable and useful tool in physiotherapy practice both as a screening tool for yellow flags and also as tool to guide and assist the level of treatment for LBP patients.

## Abbreviations

CI: Confidence interval
GP: General practices
Hannover: Hannover functional ability questionnaire
ICC: Intraclass correlation coefficient
LBP: Low back pain
PT: Physiotherapist
SBT: Keele start back tool
SD: Standard deviation
SDC: Smallest detectable change
SEM: Standard error of measurement
VAS: Visual analogue scale
α: Cronbach’s alpha
